# The relationship between homocysteine and cardiopulmonary exercise testing in patients with acute coronary syndrome after percutaneous coronary intervention

**DOI:** 10.1186/s12872-022-02976-0

**Published:** 2023-01-06

**Authors:** Jun-Ting Luo, Chun-Mei Zeng, Yan-Mei Zhao, Zhi-Yu Zeng

**Affiliations:** 1 Department of Cardiology, The First Affiliated Hospital of Guangxi Medical University, Nanning, Guangxi China; 2 Guangxi Key Laboratory of Precision Medicine in Cardio-Cerebrovascular Diseases Control and Prevention and Guangxi Clinical Research Center for Cardio-Cerebrovascular Diseases, Nanning, Guangxi China; 3 Department of Cardiology, The Sixth Affiliated Hospital, Guangxi Medical University, Yulin, Guangxi China

**Keywords:** Homocysteine, Acute coronary syndrome, Cardiopulmonary exercise testing

## Abstract

**Objective:**

The purpose of this study was to investigate the relationship between homocysteine (Hcy) levels and cardiopulmonary exercise testing (CPET) in patients with acute coronary syndrome (ACS) after percutaneous coronary intervention (PCI). We also explored the relationship between Hcy levels and cardiac ultrasonography.

**Methods:**

This study comprised 261 patients with ACS who underwent coronary angiography and PCI at Yulin First Hospital from January 2020 to June 2021. All subjects completed basic data collection, laboratory examination, CPET and cardiac ultrasonography. The CPET includes the peak oxygen uptake (peak VO_2_), anaerobic threshold (AT), metabolic equivalents (METs), exercise load (load), oxygen pulse (O_2_ pulse), end-tidal CO_2_ partial pressure (PETCO_2_), ventilatory equivalents for carbon dioxide (VE/VCO_2_) and Oxygen uptake efficiency (OUES). Cardiac ultrasonography was used to evaluate the left ventricular end diastolic diameter (LVEDD), interventricular septal thickness (IVST), left ventricular posterior wall thickness (LVPWT) and left ventricular ejection fraction (LVEF). A serum Hcy level ≥ 15 µmol/L was defined as hyperhomocysteinemia (HHcy). The patients were divided into the Hcy < 15 µmol/L group (n = 189) and the Hcy ≥ 15 µmol/L group (n = 72).

**Results:**

The average age of the participating patients was 58.9 ± 10.1 years. The majority of participants were male (86.6%). The CPET indices of METs, load, VO_2_/kg, and PETCO_2_ were significantly decreased in the Hcy ≥ 15 µmol/L group compared with the Hcy < 15 µmol/L group. Additionally, the CPET index of the VE/VCO_2_ slope and the cardiac ultrasonography indices of IVST and LVPWT were significantly increased in the Hcy ≥ 15 µmol/L group compared with the Hcy < 15 µmol/L group. These differences were statistically significant (*P* < 0.05). Correlation analysis showed that Hcy levels were negatively correlated with METs, VO_2_/kg and PETCO_2_ and positively correlated with the VE/VCO_2_ slope (*P* < 0.05). Partial correlation analysis showed that Hcy levels were negatively correlated with METs and VO_2_/kg in the AT state. The correlation coefficients were − 0.172 and − 0.172, respectively (*P* < 0.05). Hcy levels were negatively correlated with METs, VO_2_/kg and PETCO_2_ in the peak state. The correlation coefficients were − 0.177, -0.153 and − 0.129, respectively (*P* < 0.05). After further adjustment for confounders, multiple linear regression analysis showed that Hcy levels were negatively correlated with METs and VO_2_/kg in the AT state and peak state. The standardized regression coefficients were − 0.035, -0.122, -0.048 and − 0.128, respectively (*P* < 0.05). Correlation analysis showed that Hcy levels were positively correlated with the IVST and LVPWT (*P* < 0.05), but after adjusting for confounding factors, partial correlation analysis showed that there was no correlation between them.

**Conclusion:**

A high Hcy level is associated with lower METs and VO_2_/kg and worse cardiopulmonary function in patients with ACS after PCI.

## Introduction

Acute coronary syndrome (ACS) is a common cardiovascular emergency that includes unstable angina pectoris (UAP), non-ST elevated myocardial infarction (NSTEMI) and ST elevated myocardial infarction (STEMI). It has an acute onset, rapid progression and a high mortality rate, which endangers the lives, health and safety of people [1]. PCI is the best treatment for patients with blocked blood vessels. After PCI treatment, the blood and oxygen supply of patients is improved to a certain extent, which can lead to improved motor function. Through cardiac rehabilitation treatment, the cardiopulmonary function of patients can be greatly improved.

Cardiopulmonary exercise testing (CPET) is a noninvasive test method to evaluate cardiopulmonary function and exercise endurance. It can objectively and comprehensively evaluate cardiopulmonary function, which cannot be done by traditional equipment. During CPET, measures of pulmonary ventilation (VE), oxygen uptake (VO_2_) and carbon dioxide output (VCO_2_) can be obtained. Then, we can calculate indices such as the peak oxygen uptake (peak VO_2_), anaerobic threshold (AT), metabolic equivalents (METs), exercise load (load), oxygen pulse (O_2_ pulse), end-tidal CO2 partial pressure (PETCO_2_), ventilatory equivalents for carbon dioxide (VE/VCO_2_) and the oxygen uptake efficiency (OUES) [2]. VO_2_ measured at peak and AT exercise are the most important indices to evaluate the patient’s cardiopulmonary function. We used peak VO_2_ and AT VO_2_ to evaluate cardiac function grading and exercise tolerance. It can also reflect the severity of heart failure (HF), coronary heart disease (CHD), pulmonary hypertension and cardiomyopathy [3, 4]. The AT values represent the moment at which anaerobic metabolism increases. AT is not affected by the subjective factors of patients. It is usually used to formulate the exercise intensity in an individual exercise prescription and evaluate the degree of cardiac function damage in combination with peak VO_2_ [5]. METs are the oxygen consumption required to maintain resting metabolism, and it is measured by peak VO_2_ using exercise tests. The exercise intensity load is calculated according to METs to guide the exercise program for patients. In the process of treadmill movement, the load increases with increasing oxygen consumption. We can calculate the exercise load (load) at peak and AT. The O_2_ pulse is the ratio of the VO_2_ and heart rate (O_2_/HR), which depends on cardiac output (CO) and the arteriovenous oxygen difference (A-VO_2_Diff). The O_2_ pulse plays an important role in the diagnosis of myocardial ischaemia [4, 6]. PETCO_2_ can evaluate ventilation perfusion and cardiac function to reflect the disease severity of patients with HF, cardiomyopathy, pulmonary hypertension, chronic obstructive ventilatory dysfunction (COPD) and other pulmonary ventilation disorders [3, 4, 7]. The VE/VCO_2_ slope is an important index of ventilatory efficiency and the severity of systemic disease [8]. In CPET, the VE/VCO_2_ slope is negatively correlated with ventilation efficiency. It is related to age and ventilation. The more severe the degree of heart failure is, the higher the value of the VE/VCO_2_ slope [9–11]. The OUES is not affected by subjective factors and are related to the severity of HF [12]. In conclusion, CPET is important in the diagnosis and treatment of cardiovascular and respiratory diseases.

Clinically, left ventricular hypertrophy (LVH) is a strong risk factor for the occurrence and development of cardiovascular disease, and it has been a focal point for cardiovascular physicians. LVH is highly prevalent in patients with ischaemic heart disease and increases the risk of myocardial infarction [13, 14]. The left ventricular end diastolic diameter (LVEDD), interventricular septal thickness (IVST), left ventricular posterior wall thickness (LVPWT) and left ventricular ejection fraction (LVEF) indices can be obtained through cardiac ultrasonography, and then, the cardiac function and LVH can be further evaluated.

Previous studies have shown that higher levels of homocysteine (Hcy) are associated with a higher risk of cardiovascular and cerebrovascular diseases [15]. Abnormal homocysteine metabolism leads to hyperhomocysteinemia (HHcy). There is no absolute limit to the homocysteine level in the clinic, but a level of homocysteine of more than 15 µmol/L is defined as HHcy [16]. This can lead to the development of atherosclerosis and further lead to ACS [17]. The possible mechanisms of action may include vascular endothelial cell damage and a decrease in the synthesis of high-density lipoprotein [18]. A recent study on ACS in individuals ≤ 35 years old showed that HHcy was significantly associated with ACS. Moreover, it has an effect on the severity of coronary artery stenosis [19]. In another study, HHcy was related to the prognosis of ACS in Chinese patients. The all-cause mortality of ACS increases with increasing levels of plasma Hcy [20]. CPET and LVH play important roles in evaluating cardiovascular disease. However, the mechanism is not fully clear. More importantly, the relationship between Hcy and CPET is still unclear. Therefore, the primary objective of this study was to evaluate the relationship between Hcy and CPET to provide important information for clinical risk assessment and to investigate new opportunities for disease prevention and treatment.

## Methods

### Study population

In this single-centre observational study, 261 patients diagnosed with ACS aged 20 to 80 years who underwent coronary angiography (CAG) and PCI at the Sixth Affiliated Hospital of Guangxi Medical University from January 2020 to June 2021 were selected. They were divided into two groups according to their concentration of Hcy. One of the groups was Hcy < 15 µmol/L (n = 189), and the other group was Hcy ≥ 15 µmol/L (n = 72). All hospital in-patients were routinely screened for Severe Acute Respiratory Syndrome Coronavirus 2 (SARS-Cov2) on admission to hospital. All the patients we included were SARS-Cov2 negative.

The exclusion criteria were as follows: (1) incomplete medical records (for example, missing homocysteine data); (2) severe renal insufficiency (end-stage renal disease requiring chronic dialysis); (3) pernicious anaemia, myocarditis, cardiomyopathy, valvular heart disease, congenital heart disease, rheumatic heart disease, malignant tumours, a recent history of surgery or trauma, pregnancy, and patients under 18 years of age; and (4) vitamin or folate supplementation within 3 months.

This study was approved by the Ethics Committee of the Sixth Affiliated Hospital of Guangxi Medical University. All patients provided written informed consent to participate in this study. This study complied with the principles of the Declaration of Helsinki for investigations involving human beings.

### Data collection and related definitions

#### General information

The basic information of each patient at admission was recorded in detail. These include age, sex, body mass index (BMI), smoking, diabetes, primary hypertension, hyperlipidaemia and chronic renal failure. We also collected laboratory indicators such as plasma levels of Hcy, triglycerides (TG), total cholesterol (TC), low-density lipoprotein cholesterol (LDL-C), high-density lipoprotein cholesterol (HDL-C), D-dimer, PT, Fibrinogen, uric acid (UA) and serum creatinine (Cr).

#### Serum Hcy measurement

First of all, Venous blood was drawn from the subjects in the fasting state (≥ 10 h). Hcy was determined by enzymatic colorimetry (DiaSys). The examination was conducted using Roche Cobas C 701/702 automatic biochemical analyzer (China, Roche Diagnostics) and Hcy test kit (China, Roche Diagnostics). The detection process and results were judged in strict accordance with the kit instructions by an experienced laboratory physician. All parameters of the instrument shall be kept constant during measurement.

### CPET

CPET was detected by MasterScreen CPX tester from German yeger. The procedures of CPET include: (1) patient preparation: the patient should wear loose and comfortable sports shoes, socks and clothing. Avoid full meals or long periods of fasting and smoking or drinking strong coffee before exercise before exercise. (2) Sign the informed consent: patients were informed of the purpose, significance, exercise plan, potential risks and normal reactions during exercise of CPET, and sign the informed consent. (3) Test preparation: inform the patient of the pedal speed (60r/min), adjust the height of the bicycle seat and handle to the optimal position, common reasons for stopping the movement and nonverbal communication methods that are obviously inappropriate during the movement. (4) Evaluation of the trial physician: according to the different severity of the patient’s disease, combined with gender, age and functional state, select the appropriate power increasing rate, and complete the maximum extreme exercise in about 10 min. Stop exercise in case of one of the following conditions: chest pain, dyspnea, pale face, fatigue, dizziness, lower limb pain and instability; ECG indicates myocardial ischemia; Severe arrhythmia; Systolic blood pressure > 250 mmHg, diastolic blood pressure > 115 mmHg or systolic blood pressure drop > 20 mmHg.

CPET was arranged when the patient’s condition was stable after PCI and informed consent was obtained. Subjects wore appropriate masks and were connected to ECG monitors to dynamically monitor ECG, blood pressure and oxygen saturation. CPET was performed on a power bike, and AT was measured by the v-slope method. The changes in oxygen consumption, blood pressure, exercise load, pulmonary ventilation index and ECG were continuously monitored during exercise. Thus, the peak VO_2_, O_2_ pulse, AT, PETCO_2_, METs, exercise load, VE/VCO_2_ slope and OUES were further calculated.

#### Cardiac ultrasonography

Patients underwent colour Doppler ultrasound during their hospitalization. Cardiac ultrasonography was used to evaluate the LVEDD, IVST, LVPWT and LVEF.

### Statistical analysis

The survey data are entered by EpiData V3.1 software. Statistical software SPSS 19.0 (Chicago, USA) was used for data analysis. Accordingly, continuous variables with normal distribution were expressed as mean ± standard deviation (SD) and compared between two groups using the independent samples t-test. The count data are expressed as number of cases and percentage (%). A chi-square test was used for the comparison between groups. Correlation analysis or partial correlation analysis was performed using Spearman correlation analysis. The relationship between CPET and Hcy was analyzed by multiple linear regression. A value of *P* < 0.05 in a two-sided test was considered statistically significant.

## Results

### Baseline clinical characteristics

A total of 261 participants, including 189 Hcy < 15 µmol/L patients and 72 Hcy ≥ 15 µmol/L patients, were enrolled in the study. Clinical characteristics and biochemical findings of involved participants are listed in Table 1. The average age of participating patients was 58.9 ± 10.1 years. The majority of participants were male (86.6%). The higher prevalence of hypertension in the Hcy ≥ 15 µmol/L group compared to the Hcy < 15 µmol/L group (66.7% vs. 48.1%, *P* < 0.05). There were no significant differences in age, sex, BMI, smoking history, hyperlipidemia, diabetes mellitus, triglyceride, total cholesterol, D-dimer, PT, Fibrinogen, resting systolic blood pressure (SBP), resting diastolic blood pressure (DBP) and resting heart rate (HR) between the two groups (*P* > 0.05). There were a significant difference in the creatinine, uric acid and plasma Hcy level between the two groups (*P* < 0.05). We found that all the patients have TIMI3 flow after PCI. In our study, 70.9% were STEMI patients and 29.1% were NSTEMI patients. There were more NSTEMI patients and in the Hcy ≥ 15 µmol/L group compared to the Hcy < 15 µmol/L group, but there were no difference between the two groups (*P* > 0.05). According to the angiographic data, we further analyzed the culprit vessel and there were no difference between the two groups (*P* > 0.05).

**Table 1:** Baseline clinical characteristics of study participants

Characteristics	Participants (n = 261)	Hcy < 15 µmol/L (n = 189)	Hcy ≥ 15µmol/L (n = 72)	*P* value
Age (years)	58.9 ± 10.1	58.2 ± 9.5	60.8 ± 11.4	0.063
Male [n (%)]	226 (86.6)	159 (84.1)	67 (93.1)	0.058
Smoker [n (%)]	138 (52.9)	100 (52.9)	38 (52.8)	0.985
BMI (kg/m^2^)	24.25 ± 2.76	24.28 ± 2.70	24.19 ± 2.91	0.813
Total cholesterol (mmol/L)	4.53 ± 1.07	4.59 ± 1.10	4.38 ± 0.96	0.173
Triglyceride (mmol/L)	1.67 ± 0.97	1.73 ± 1.01	1.54 ± 0.84	0.119
Serum creatinine (µmol/L)	90.5 ± 26.1	84.87 ± 18.9	105.4 ± 35.2	< 0.001
Uric acid (µmol/L)	368.8 ± 102.6	359.8 ± 97.6	392.7 ± 111.9	0.020
D-dimer	0.67 ± 0.76	0.63 ± 0.76	0.77 ± 0.75	0.197
PT (s)	11.49 ± 1.38	11.46 ± 1.18	11.58 ± 1.80	0.549
Fibrinogen	4.01 ± 1.60	3.99 ± 1.57	4.06 ± 1.70	0.753
hypertension [n (%)]	139 (53.3)	91 (48.1)	48 (66.7)	0.007
Diabetes [n (%)]	62 (23.8)	47 (24.9)	15 (20.8)	0.494
hyperlipidemia [n (%)]	96 (36.8)	76 (40.2)	20 (27.8)	0.063
ACS type [n (%)]				0.066
STEMI [n (%)]	185 (70.9)	140 (74.1)	45 (62.5)	
NSTEMI [n (%)]	76 (29.1)	49 (25.9)	27 (37.5)	
Culprit vessel				0.119
LAD [n (%)]	139 (53.3)	107 (56.6)	32 (44.4)	
LCx [n (%)]	50 (19.1)	31 (16.4)	19 (26.4)	
RCA [n (%)]	72 (27.6)	51 (27.0)	21 (29.2)	
Resting SBP (mmHg)	111.3 ± 16.8	110.7 ± 17.1	112.8 ± 16.2	0.355
Resting DBP (mmHg)	72.0 ± 11.4	71.2 ± 11.0	74.1 ± 12.3	0.069
Resting HR (bpm)	76.0 ± 12.9	75.5 ± 12.8	77.1 ± 12.9	0.398
Hcy (µmol/L)	13.08 ± 3.60	11.37 ± 2.03	17.57 ± 2.89	< 0.001

*BMI* body mass index,* Hcy* homocysteine,* PT* prothrombin time,* ACS* acute coronary syndrome,* STEMI* ST elevated myocardial infarction,* NSTEMI* non-ST elevated myocardial infarction,* LAD* left anterior descending,* LCx* left circumflex,* RCA* right coronary artery,* SBP* systolic blood pressure,* DBP* diastolic blood pressure,* HR* heart rate

### Comparison of Hcy with CPET and cardiac ultrasonography

Compared with the Hcy < 15 µmol/L group, patients with Hcy ≥ 15 µmol/L showed a significant decrease in the CPET index (METs, Load, VO_2_/kg, PETCO_2_). while showed a significant increase in the CPET index (VE/VCO_2_ slope) and cardiac ultrasonography (IVST, LVPWT). The difference was statistically significant (*P* < 0.05, Table 2).

**Table 2:** Comparison of CPET, Cardiac Ultrasonography between Hcy < 15 µmol/L group and Hcy ≥ 15 µmol/L group

	Hcy < 15 µmol/L (n = 189)	Hcy ≥ 15 µmol/L(n = 72)	*P* value
AT			
METs	4.25 ± 0.92	3.92 ± 0.85	0.009
Load (w)	74.1 ± 20.6	67.3 ± 18.5	0.015
VO_2_/kg [ mL/(min kg)]	14.87 ± 3.24	13.72 ± 2.97	0.009
O_2_ pulse (mL/beat)	8.92 ± 2.10	8.54 ± 2.06	0.194
PETCO_2_	31.27 ± 4.28	30.02 ± 3.45	0.028
Peak			
METs	5.18 ± 1.14	4.70 ± 0.99	0.002
Load (w)	94.84 ± 25.06	86.28 ± 19.92	0.005
VO_2_/kg [ mL/(min kg)]	18.20 ± 3.86	16.67 ± 3.14	0.003
O_2_ pulse (mL/beat)	9.97 ± 2.32	9.52 ± 2.04	0.154
PETCO_2_	30.45 ± 4.34	28.88 ± 3.56	0.007
VE/VCO_2_ slope	35.56 ± 5.77	37.32 ± 6.49	0.034
OUES	1402.20 ± 364.58	1339.42 ± 368.27	0.216
Cardiac ultrasonography			
IVST (mm)	11.00 ± 1.61	11.54 ± 1.62	0.018
LVPWT (mm)	10.88 ± 1.53	11.39 ± 1.44	0.016
LVEDD (mm)	46.39 ± 4.50	47.31 ± 6.42	0.268
LVEF (%)	56.17 ± 9.31	55.64 ± 10.63	0.690

*Hcy* homocysteine,* AT* anaerobic threshold,* METs* metabolic equivalents,* O*_2_ pulse oxygen pulse,* PETCO*_2_ End-tidal CO2 partial pressure,* VE/VCO*_2_ Ventilatory equivalents for carbon dioxide,* OUES* oxygen uptake efficiency,* IVST* interventricular septal thickness,* LVPWT* left ventricular posterior wall thickness,* LVEDD* left ventricular end diastolic diameter,* LVEF* left ventricular ejection fraction

#### Correlation analysis of Hcy with CPET and cardiac ultrasonography

**Fig. 1:**
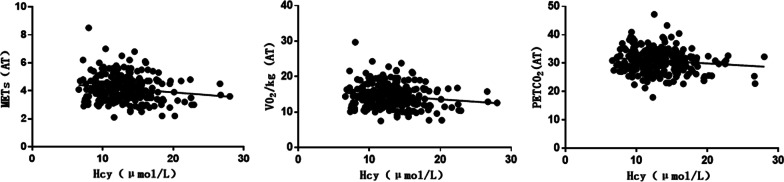
Correlation between Hcy and (METs, Load, VO_2_/kg, PETCO_2_) in AT

**Fig. 2:**
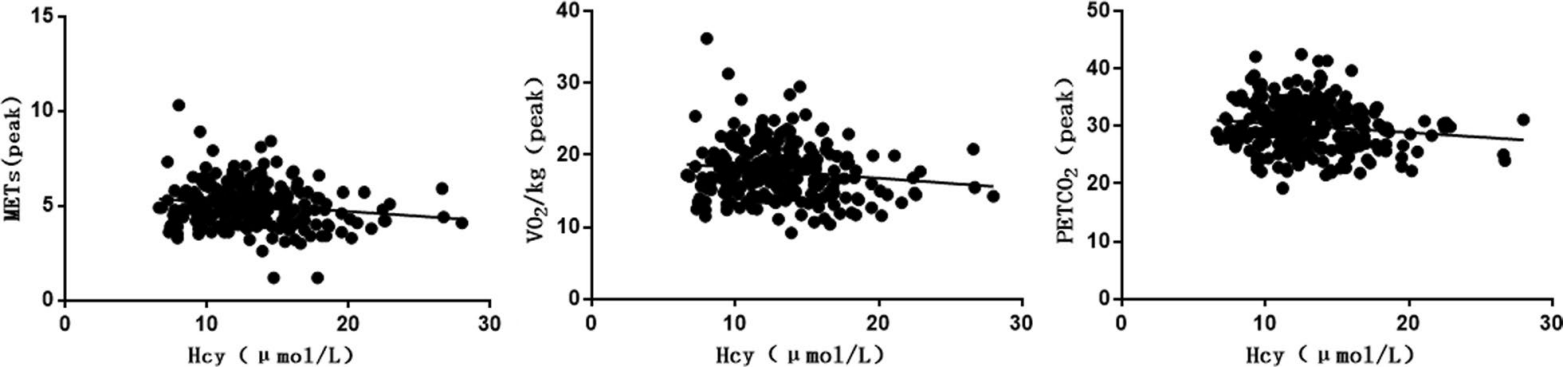
Correlation between Hcy and (METs, load, VO_2_/kg, PETCO_2_) in peak

**Fig. 3:**
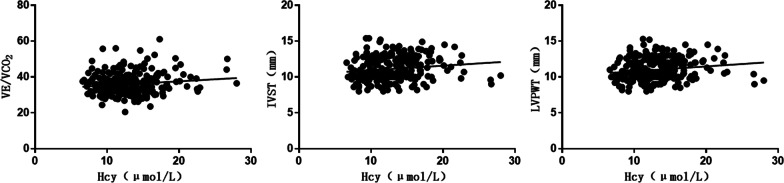
Correlation between Hcy and VE/VCO_2_ slope, IVST and LVPWT

 Correlation analysis showed that Hcy correlated negatively with (METs, VO_2_/kg, PETCO_2_) and positively with VE/VCO_2_ slope, IVST and LVPWT. The difference was statistically significant (*P* < 0.05, Table 3; Figs. 1, 2 and 3).

**Table 3:** Correlation analysis of Hcy with CPET and cardiac ultrasonography

	*r*	*P* value
AT		
METs	− 0.152	0.014
Load (w)	− 0.101	0.103
VO_2_/kg [ mL/(min kg)]	− 0.152	0.014
O_2_ pulse (mL/beat)	− 0.029	0.636
PETCO_2_	− 0.126	0.043
Peak		
METs	− 0.160	0.009
Load (w)	− 0.079	0.202
VO_2_/kg [ mL/(min kg)]	− 0.139	0.024
O_2_ pulse (mL/beat)	− 0.027	0.663
PETCO_2_	− 0.137	0.026
VE/VCO_2_ slope	0.136	0.028
OUES	0.010	0.878
Cardiac ultrasonography		
IVST (mm)	0.142	0.023
LVPWT (mm)	0.162	0.009
LVEDD (mm)	0.090	0.148
LVEF (%)	-0.007	0.905

*AT* anaerobic threshold,* METs* metabolic equivalents,* O*_2_ pulse oxygen pulse,* PETCO*_2_ End-tidal CO2 partial pressure,* VE/VCO*_2_ Ventilatory equivalents for carbon dioxide,* OUESs* oxygen uptake efficiency,* IVST* interventricular septal thickness,* LVPWT* left ventricular posterior wall thickness,* LVEDD* left ventricular end diastolic diameter,* LVEF* left ventricular ejection fraction

#### Relationship between CPET, cardiac ultrasonography and Hcy

After adjusting for age, sex and BMI, partial correlation analysis showed that Hcy was negatively correlated with (METs, VO_2_/kg) in AT state. The correlation coefficients were − 0.172 and − 0.172 (*P* < 0.05). Hcy was negatively correlated with (METs, VO_2_/kg, PETCO_2_) in peak state. The correlation coefficients were − 0.177, − 0.153 and − 0.129 (*P* < 0.05). Hcy was positively with VE/VCO_2_ slope, IVST and LVPWT. But the difference is not statistically significant (*P* > 0.05). Multiple linear regression analysis was performed with (METs, VO_2_/kg, PETCO_2_) as independent variables and Hcy as dependent variables. Model 1 no factors were adjusted; Model 2 adjusts for age, sex, BMI, hypertension, diabetes, hyperlipidemia, uric acid and creatinine. After further adjustment for confounders (model 2), Hcy was negatively correlated with (METs, VO_2_/kg) in AT state and peak state. The standardized regression coefficients were − 0.035, − 0.122, − 0.048 and − 0.128 respectively. (*P* < 0.05, Table 4).

**Table 4:** Relationship between CPET, cardiac ultrasonography and Hcy

	*r*	Multiple linear regression	
		Model 1 (*β*)	Model 2 (*β*)
AT			
METs	− 0.172**	− 0.039^*^	− 0.035^*^
VO_2_/kg [ mL/(min kg)]	− 0.172**	− 0.135^*^	− 0.122^*^
PETCO_2_	− 0.109^#^	–	–
Peak			
METs	− 0.177**	− 0.050^**^	− 0.048^*^
VO_2_/kg [ mL/(min kg)]	− 0.153*	− 0.145^*^	− 0.128^*^
PETCO_2_	− 0.129*	− 0.160^*^	− 0.111
VE/VCO2 slope	0.113^#^	–	–
IVST (mm)	0.100	–	–
LVPWT (mm)	0.113^#^	–	–

*Hcy* homocysteine,* AT* anaerobic threshold,* METs* metabolic equivalents,* PETCO*_2_ End-tidal CO2 partial pressure,* VE/VCO*_2_ Ventilatory equivalents for carbon dioxide,* IVST* interventricular septal thickness,* LVPWT* left ventricular posterior wall thickness. Model 1 no factors were adjusted; Model 2 adjusts for age, sex, BMI, hypertension, diabetes, hyperlipidemia, uric acid and creatinine

^#^*P*＜0.1; *P＜0.05;  **P＜0.01

## Discussion

Hcy is a sulfur-containing amino acid metabolite that is synthesized in the process of methionine metabolism. A high Hcy level can lead to cardiovascular and cerebrovascular diseases. Studies have reported that an elevated level of Hcy is a risk factor for CHD, stroke and hypertension [15, 21]. HHcy is an important indicator for the evaluation of human health, especially for hypertension, hyperlipidaemia and hyperglycaemia. A systematic review of HHcy in China found that the prevalence of high Hcy was 27.5% [22]. HHcy diagnostic criteria are defined as Hcy ≥ 15 µmol/L. Therefore, we used 15 µmol/L as the cut-off value for grouping patients according to Hcy levels for further research. The Hcy level is related to 30-day cardiovascular events in patients with ACS [23]. In our study, we analysed the clinical baseline data of 261 patients with ACS after PCI. There is still no consistent opinion about the relationship between the Hcy level and long-term outcomes of ACS patients. We found that, compared with patients in the Hcy < 15 µmol/L group, patients in the Hcy ≥ 15 µmol/L group had a higher prevalence of hypertension and higher levels of creatinine and uric acid. These findings are consistent with previous studies [24–28]. In addition, HHcy increased with age and was higher in men than in women.

CHD seriously threatens human health and greatly increases the risk of death. ACS is even more fatal. Previous studies have shown that CPET is helpful in the diagnosis of CHD [10, 29]. International guidelines on CPET also put forward the importance of the CPET index in the diagnosis of CHD [8]. ECG, blood pressure and heart rate can be monitored during CPET. Another study showed that AT and peak oxygen uptake in patients with CHD were significantly lower than in those without CHD [30]. CPET can detect the cardiopulmonary function reserve and the degree of functional impairment of patients and help understand the gas exchange rate of patients [31]. The worse the cardiopulmonary function is, the worse the prognosis. Previous studies have suggested that CPET is of great significance in the assessment of cardiovascular, respiratory, and metabolic diseases[2, 32]. However, there is no relevant study on Hcy level and CPET. This study was the first time to propose that the correlations between Hcy level and CPET.

In the process of the CPET, a gradual increase in the exercise load should gradually increase the cardiac output. The degree of myocardial ischaemia and prognosis can be detected and evaluated through CPET. The prognosis of patients with CHD can be improved by cardiac rehabilitation according to cardiac ultrasonography and a quality of life scale. However, it cannot replace the accuracy and objectivity of CPET indicators. By evaluating AT, peak VO_2_ and other related indicators, we can formulate the best indicators of aerobic exercise prescription [8, 33]. We hypothesize that high Hcy levels may affect the cardiopulmonary function of ACS patients. Our study confirms this hypothesis. In the present study, we found that, compared with the Hcy ≥ 15 µmol/L group, the Hcy < 15 µmol/L group had better cardiopulmonary function. Hcy was negatively correlated with METs and VO_2_/kg in the AT state and peak state. We speculate that the possible mechanism by which Hcy aggravates the damage to cardiopulmonary function in patients with ACS is that Hcy promotes myocardial hypertrophy and damages myocardial systolic function through oxidative reactions and the activation of sympathetic nerves. Hcy-lowering interventions should be considered to prevent cardiovascular events [34] and improve cardiopulmonary function. Folic acid and B vitamins can reduce Hcy levels [35, 36].

Previous studies have found that Hcy levels are associated with cardiac systolic function in patients with CAD [37]. With an increase in the Hcy level, echocardiographic indices, such as left ventricular (LV) hypertrophy and cardiac diastolic dysfunction, could change in patients with obstructive sleep apnoea syndrome (OSAS) [38]. In our study, when we compared the Hcy < 15 µmol/L group and Hcy ≥ 15 µmol/L group, the IVST and LVPWT were significantly increased in the Hcy ≥ 15 µmol/L group. Correlation analysis showed that Hcy levels positively correlated with the IVST and LVPWT. However, after adjusting for confounding factors, partial correlation analysis showed that there was no correlation between these parameters. The results of this study are inconsistent with those of previous studies[39–41]. We consider that this may be related to the small sample size and the fact that some patients underwent bedside cardiac colour Doppler ultrasound. Moreover, the cardiac colour Doppler ultrasound indices of ACS patients changed greatly. Therefore, we need to conduct long-term follow-up monitoring and expand the sample size for further confirmation.

This study is the first report the relationship between Hcy levels and CPET. However, due to the limited number of patients in this study, more evidence is needed to draw a conclusion about this issue. Moreover, this study included only ACS patients after PCI. More patients with other diseases should be studied in the future. These patients can be followed up for a long time to understand the improvements in cardiopulmonary function after further treatment.

## Conclusion

There was a correlation between Hcy levels and CPET. A higher Hcy level was associated with worse cardiopulmonary function in patients with ACS after PCI. Attention should be given to blood Hcy levels in patients with ACS.

## Data Availability

The datasets generated and/or analyzed during the current study are available from the corresponding author on reasonable request.

## Abbreviations

Hcy: Homocysteine
HHCY: Hyperhomocysteinemia
CPET: Cardiopulmonary exercise testing
ACS: Acute coronary syndrome
PCI: Percutaneous coronary intervention
PT: Prothrombin time
LAD: Left anterior descending
LCx: Left circumflex
RCA: Right coronary artery
AT: Anaerobic threshold
METs: Metabolic equivalents
O_2_ pulse: Oxygen pulse
PETCO_2_: End-tidal CO_2_ partial pressure
VE/VCO_2_: Ventilatory equivalents for carbon dioxide
OUES: Oxygen uptake efficiency
VE: Ventilation
VO_2_: Oxygen uptake
VCO_2_: Carbon dioxide output
peak VO_2_: Peak oxygen uptake
HF: Heart failure
CHD: Coronary heart disease
CO: Cardiac output
A-VO_2_Diff: Arteriovenous oxygen difference
LVEDD: Left ventricular end diastolic diameter
IVST: Interventricular septal thickness
LVPWT: Left ventricular posterior wall thickness
LVEF: Left ventricular ejection fraction
UAP: Unstable angina pectoris
NSTEMI: Non-ST elevated myocardial infarction
STEMI: ST elevated myocardial infarction
COPD: Chronic obstructive ventilatory dysfunction
LVH: Left ventricular hypertrophy
CAG: Underwent coronary angiography
BMI: Body mass index
TG: Triglycerides
TC: Total cholesterol
LDL-C: Low-density lipoprotein cholesterol
HDL-C: high-density lipoprotein cholesterol
UA: Uric acid
Cr: Serum creatinine
SBP: Systolic blood pressure
DBP: Diastolic blood pressure
HR: Heart rate
OSAS: Obstructive sleep apnea syndrome.

## References

[1] Smith JN, Negrelli JM, Manek MB, Hawes EM, Viera AJ (2015). Diagnosis and management of acute coronary syndrome: an evidence-based update. J Am Board Fam Med.

[2] Herdy AH, Ritt LE, Stein R, Araujo CG, Milani M, Meneghelo RS, Ferraz AS, Hossri C, Almeida AE, Fernandes-Silva MM (2016). Cardiopulmonary exercise test: background, applicability and interpretation. Arq Bras Cardiol.

[3] Sorajja P, Allison T, Hayes C, Nishimura RA, Lam CS, Ommen SR (2012). Prognostic utility of metabolic exercise testing in minimally symptomatic patients with obstructive hypertrophic cardiomyopathy. Am J Cardiol.

[4] Guazzi M, Adams V, Conraads V, Halle M, Mezzani A, Vanhees L, Arena R, Fletcher GF, Forman DE, Kitzman DW (2012). EACPR/AHA Scientific Statement. Clinical recommendations for cardiopulmonary exercise testing data assessment in specific patient populations. Circulation.

[5] Agostoni P, Corra U, Cattadori G, Veglia F, Battaia E, La Gioia R, Scardovi AB, Emdin M, Metra M, Sinagra G (2013). Prognostic value of indeterminable anaerobic threshold in heart failure. Circ Heart Fail.

[6] Sociedade Brasileira de C (2010). III guidelines of Sociedade Brasileira de Cardiologia on the exercise test. Arq Bras Cardiol.

[7] Torchio R, Guglielmo M, Giardino R, Ardissone F, Ciacco C, Gulotta C, Veljkovic A, Bugiani M (2010). Exercise ventilatory inefficiency and mortality in patients with chronic obstructive pulmonary disease undergoing surgery for non-small-cell lung cancer. Eur J Cardiothorac Surg.

[8] Balady GJ, Arena R, Sietsema K, Myers J, Coke L, Fletcher GF, Forman D, Franklin B, Guazzi M, Gulati M (2010). Clinician’s Guide to cardiopulmonary exercise testing in adults: a scientific statement from the American Heart Association. Circulation.

[9] Arena R, Myers J, Guazzi M (2008). The clinical and research applications of aerobic capacity and ventilatory efficiency in heart failure: an evidence-based review. Heart Fail Rev.

[10] Wasserman K (2012). Principles of exercise testing and interpretation: including pathophysiology and clinical applications.

[11] Mezzani A, Giordano A, Komici K, Corra U (2017). Different determinants of ventilatory inefficiency at different stages of reduced ejection Fraction Chronic Heart failure natural history. J Am Heart Assoc.

[12] Davies LC, Wensel R, Georgiadou P, Cicoira M, Coats AJ, Piepoli MF, Francis DP (2006). Enhanced prognostic value from cardiopulmonary exercise testing in chronic heart failure by non-linear analysis: oxygen uptake efficiency slope. Eur Heart J.

[13] Frey N, Katus HA, Olson EN, Hill JA (2004). Hypertrophy of the heart: a new therapeutic target?. Circulation.

[14] Stewart MH, Lavie CJ, Shah S, Englert J, Gilliland Y, Qamruddin S, Dinshaw H, Cash M, Ventura H, Milani R (2018). Prognostic implications of left ventricular hypertrophy. Prog Cardiovasc Dis.

[15] Liu J, Quan J, Li Y, Wu Y, Yang L (2018). Blood homocysteine levels could predict major adverse cardiac events in patients with acute coronary syndrome: a STROBE-compliant observational study. Med (Baltim).

[16] Hankey GJ, Eikelboom JW (1999). Homocysteine and vascular disease. Lancet.

[17] Zhao J, Chen H, Liu N, Chen J, Gu Y, Chen J, Yang K (2017). Role of hyperhomocysteinemia and hyperuricemia in pathogenesis of atherosclerosis. J Stroke Cerebrovasc Dis.

[18] Xiao W, Bai Y, Ye P, Luo L, Liu D, Wu H, Bai J (2014). Plasma homocysteine is associated with aortic arterial stiffness but not wave reflection in Chinese hypertensive subjects. PLoS ONE.

[19] Sun J, Han W, Wu S, Jia S, Yan Z, Guo Y, Zhao Y, Zhou Y, Liu W (2021). Associations between hyperhomocysteinemia and the presence and severity of acute coronary syndrome in young adults ≤ 35 years of age. BMC Cardiovasc Disord.

[20] Fu Z, Qian G, Xue H, Guo J, Chen L, Yang X, Shen M, Dong W, Chen Y (2015). Hyperhomocysteinemia is an independent predictor of long-term clinical outcomes in chinese octogenarians with acute coronary syndrome. Clin Interv Aging.

[21] Huo Y, Li J, Qin X, Huang Y, Wang X, Gottesman RF, Tang G, Wang B, Chen D, He M (2015). Efficacy of folic acid therapy in primary prevention of stroke among adults with hypertension in China: the CSPPT randomized clinical trial. JAMA.

[22] Yang B, Fan S, Zhi X, Wang Y, Wang Y, Zheng Q, Sun G (2014). Prevalence of hyperhomocysteinemia in China: a systematic review and meta-analysis. Nutrients.

[23] Ma Y, Li L, Geng XB, Hong Y, Shang XM, Tan Z, Song YX, Zhao GY, Zhao BQ, Tian MR (2016). Correlation between hyperhomocysteinemia and outcomes of patients with Acute myocardial infarction. Am J Ther.

[24] Zhong F, Zhuang L, Wang Y, Ma Y (2017). Homocysteine levels and risk of essential hypertension: a meta-analysis of published epidemiological studies. Clin Exp Hypertens.

[25] Yang Q, Lu Y, Deng Y, Xu J, Zhang X (2020). Homocysteine level is positively and independently associated with serum creatinine and urea nitrogen levels in old male patients with hypertension. Sci Rep.

[26] Xie D, Yuan Y, Guo J, Yang S, Xu X, Wang Q, Li Y, Qin X, Tang G, Huo Y (2015). Hyperhomocysteinemia predicts renal function decline: a prospective study in hypertensive adults. Sci Rep.

[27] Wang B, Lin L, Zhao C (2016). Related factors of serum uric acid in patients with primary hypertension and hyperhomocysteinemia. Clin Exp Hypertens.

[28] Uehara SK, Rosa G (2008). Association of homocysteinemia with high concentrations of serum insulin and uric acid in brazilian subjects with metabolic syndrome genotyped for C677T polymorphism in the methylenetetrahydrofolate reductase gene. Nutr Res.

[29] Chaudhry S, Arena R, Wasserman K, Hansen JE, Lewis GD, Myers J, Chronos N, Boden WE (2009). Exercise-induced myocardial ischemia detected by cardiopulmonary exercise testing. Am J Cardiol.

[30] Akinci Ozyurek B, Savas Bozbas S, Aydinalp A, Bozbas H, Ulubay G (2019). Value of cardiopulmonary exercise testing in the diagnosis of coronary artery disease. Tuberk Toraks.

[31] Miki K (2021). Motor pathophysiology related to Dyspnea in COPD evaluated by cardiopulmonary exercise testing. Diagnostics (Basel).

[32] DeCato TW, Haverkamp H, Hegewald MJ (2020). Cardiopulmonary exercise testing (CPET). Am J Respir Crit Care Med.

[33] Fletcher GF, Ades PA, Kligfield P, Arena R, Balady GJ, Bittner VA, Coke LA, Fleg JL, Forman DE, Gerber TC (2013). Exercise standards for testing and training: a scientific statement from the American Heart Association. Circulation.

[34] Marti-Carvajal AJ, Sola I, Lathyris D (2015). Homocysteine-lowering interventions for preventing cardiovascular events. Cochrane Database Syst Rev.

[35] Mao X, Xing X, Xu R, Gong Q, He Y, Li S, Wang H, Liu C, Ding X, Na R (2016). Folic acid and vitamins D and B12 correlate with homocysteine in chinese patients with Type-2 diabetes mellitus, hypertension, or cardiovascular disease. Medicine (Baltimore).

[36] Wang WW, Wang XS, Zhang ZR, He JC, Xie CL (2017). A meta-analysis of folic acid in combination with anti-hypertension drugs in patients with hypertension and hyperhomocysteinemia. Front Pharmacol.

[37] Bokhari SW, Bokhari ZW, Zell JA, Lee DW, Faxon DP (2005). Plasma homocysteine levels and the left ventricular systolic function in coronary artery disease patients. Coronary Artery Dis.

[38] Sariman N, Levent E, Aksungar FB, Soylu AC, Bektas O (2010). Homocysteine levels and echocardiographic findings in obstructive sleep apnea syndrome. Respiration.

[39] Zheng H, Li Y, Xie N, Xu H, Huang J, Luo M (2014). Echocardiographic assessment of hypertensive patients with or without hyperhomocysteinemia. Clin Exp Hypertens.

[40] Joseph J, Joseph L, Shekhawat NS, Devi S, Wang J, Melchert RB, Hauer-Jensen M, Kennedy RH (2003). Hyperhomocysteinemia leads to pathological ventricular hypertrophy in normotensive rats. Am J Physiol Heart Circ Physiol.

[41] Lin BY, Li P, Wu XD, Li H, Zeng ZY (2020). The relationship between homocysteine, blood pressure variability, and left ventricular hypertrophy in patients with essential hypertension: an observational study. Adv Ther.

